# The Past, Present, and Future of One Health in India: A Narrative Review

**DOI:** 10.7759/cureus.44992

**Published:** 2023-09-10

**Authors:** Juhi Raut, Abhishek Joshi, Abhay Mudey, Ashok M Mehendale

**Affiliations:** 1 Community Medicine, Jawaharlal Nehru Medical College, Datta Meghe Institute of Higher Education and Research, Wardha, IND; 2 Preventive Medicine, Jawaharlal Nehru Medical College, Datta Meghe Institute of Higher Education and Research, Wardha, IND

**Keywords:** one health challenges, one health implementation, one health perspective, one health approach, one health in india

## Abstract

Humans have experienced a long-lasting pandemic of COVID-19 going on since the year 2020. Such events have recently increased the demand for a competent disease outbreak response system, more precisely, a One Health platform. The interaction between humans, animals, and ecosystems is inevitable. It is a known fact that the interface between these three entities is important for survival. In rural areas, especially in developing countries, it is a common practice to keep the animal shed in close proximity to their homes. Further, this intricate relationship itself plays a role in the spread and transmission of the disease. The involvement of the human-animal interface in emerging and re-emerging diseases has caused havoc in recent times and might prove challenging to overcome. Over the years, many efforts have been made on international and national platforms to adopt and implement a transdisciplinary, collaborative, intersectoral approach in India. This review highlights the major initiatives taken for the implementation of one health and the challenges faced over the years in our country.

## Introduction and background

Humans have experienced a long-lasting pandemic, COVID-19 going on since 2020. Such events have recently increased the demand for a competent disease outbreak response system, specifically a One Health (OH) platform. OH is a commission set up as a multisectoral, multi-disciplinary, collaborative approach working towards a Sustainable health delivery system [1]. A Holistic approach validated by international organizations, the World Health Organization (WHO), World Organization for Animal Health (OIE), and the Food and Agriculture Organization (FAO) of the United Nations to work in collaboration for the welfare of the whole ecosystem through human-animal-plant-environment interaction [2]. Communicable diseases have become a mystery due to the re-emergence of infections thought to have vanished or dominated by medicine [3]. About 350 infectious diseases, including 54% bacterial and rickettsial, were noted from 1940 to 2004. Among these, 60.3% were zoonotic diseases, and 71.8% were emerging diseases of wildlife origin [4]. Although the term OH is seldom used, its idea is not recent. Right since modern medicine, the concept of interdependent faculties for diagnosis and treatment is famous, and the use of familiar approaches like One Medicine and Eco-health is the parent concept of this approach [5].

SA has been in frontward for remerging diseases with India contributing to major chunk to its rising burden. Promoting multisectoral activities for the prevention of zoonosis and potential epidemic and pandemic are on the roll. Yet institutionalizing and functioning of various sectors related to this commission is challenging and difficult to implement [6]. Though this approach is facing difficulties in operationalization and implementation in South Asian countries, the transdisciplinary approach mandates all sectors to work together with shared responsibilities and challenges [7]. The interaction between humans, animals, and ecosystems is inevitable. It is a known fact that the interface between these three entities is important for survival. In rural areas, especially in developing countries, it is a common practice to keep the animal shed in close proximity to their homes. Further, this intricate relationship itself plays a role in the spread of the diseases. OH mandates all healthcare providers, doctors, veterinarians, cattle breeders, medical, paramedical staff, wildlife experts, and the general public to work in collaboration to provide hygienic breeding places for animals; safe food and clean water; manage antibiotic resistance, zoonotic diseases, and vector-borne infections. Hence demands a complex health system which most developing countries face challenges, due to frail structure [8]. Moreover, operationalization of such a multi-sectorial approach is challenging for both developing as well as developed countries as it demands a regional, local, national, and global level of coordination and involvement of various sectors to collaborate and work for a shared vision [9]. This review focuses on the major initiatives taken for the implementation of OH and the challenges faced over the years in our country.

## Review

Methodology

**Figure 1:**
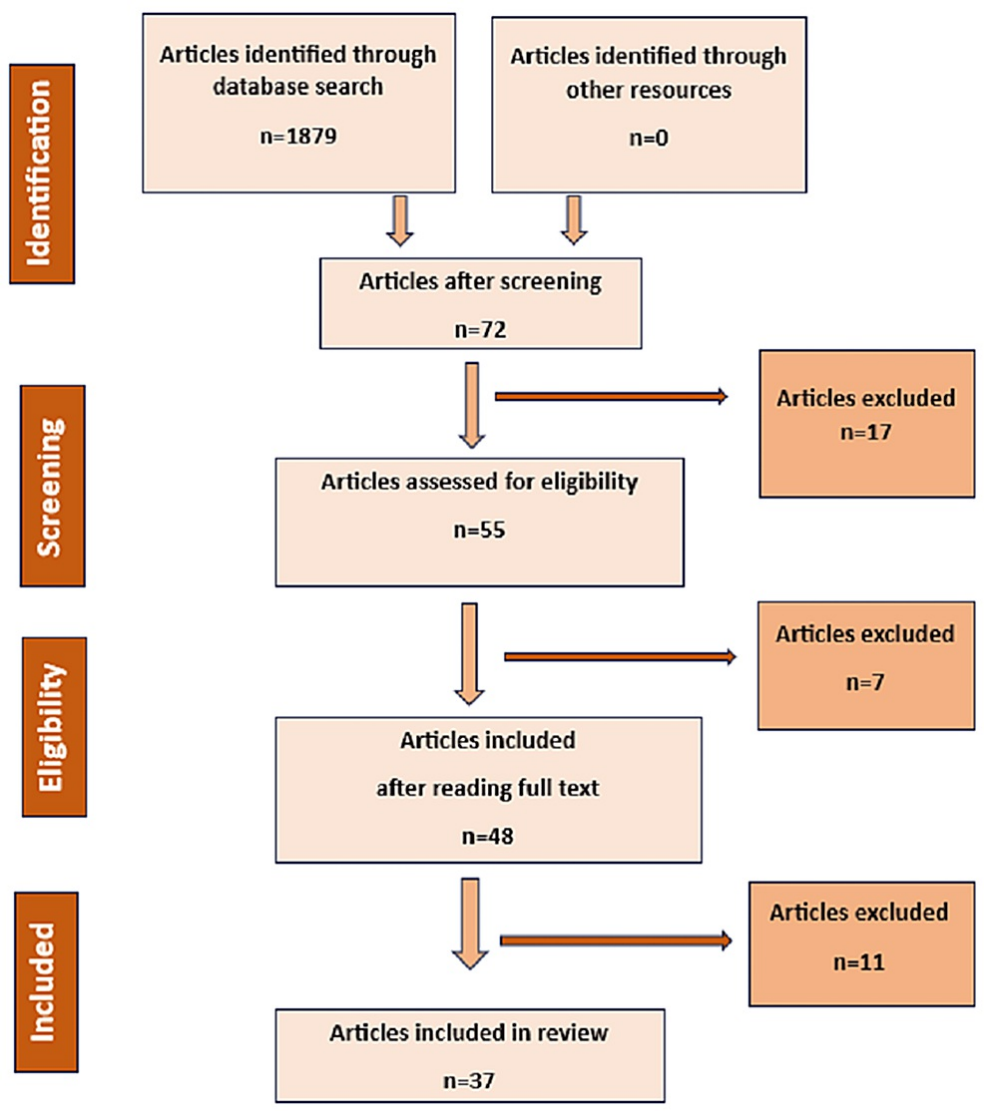
The eligibility criteria of the literature search on One Health, its implementation, operationalization and challenges in India

The eligibility criteria for this review included all articles, studies, and documents that discussed the implementation of OH, its operationalization, and challenges in India. For the literature search we used an electronic database of PubMed and Google Scholar for relevant results using the keywords; “One health in India,” ”one health approach,” “one health perspective,” “one health implementation and Challenges,” etc. These were combined with “AND”, and “OR” to obtain desired results. To timeline of 10 years from 2003 to 2023 was set. All research articles available in full text for free were included in the review. The articles from the literature search were reviewed by all authors independently. The eligibility criteria of the literature search process are shown in Figure 1.

Multisectoral aspect of OH approach

Antimicrobial Resistance (AMR)

Antimicrobial resistance (AMR) is a global public health concern because of its fast spread. India is among the top consumers of antibiotics in the world. It has the most diverse and unique socio-economic-cultural strata. The constraints faced by such a uniquely distributed population are mainly due to abuse and over-the-counter use of antibiotics, unawareness regarding antibiotic use and drug sales, and accumulation of residual in the ecosystem [10].

Major Areas Affected by Overuse of Antibiotics

Human - the highest burden of infectious diseases lies in India. Resistance to higher antibiotics has already set in for easily curable diseases hence it transformed into a public health problem. Highly resistant diseases are evolving due to improper use of antibiotics which causes multidrug-resistance (MDR) and extensively drug-resistance (XDR) tuberculosis, methicillin, and vancomycin resistant MDR Staphylococcus species.

Animal husbandry* *- overuse of antibiotics is not restricted just to man, but also to animals, to keep them healthy which ultimately will produce higher amount of milk and meat [10]. The demand for other animal products like goat, chicken, cattle, pigs, seafood, honeybees, etc. has increased recently [11]. The bioaccumulation of antibiotics in animal products may pose a threat to public health [10]. Moreover, some studies have also reported Reverse zoonosis from humans to animals, e.g., SARS‑CoV-2 [4].

Fishery - resistant species of Vibrio and Enterobacteriaceae pathogens are not only known to cause gastrointestinal infections through water bodies but also a variety of aquatic animals mainly fishes and shellfish [10]. Thus direct contact through food and animal products and the environment can also lead to the transfer of resistant antibiotics to humans [11].

To resolve the global challenge of antimicrobial resistance, there is a need to create international collaborative platforms for learning [12]. The global antibiotic resistance crisis has created a need for rational use of new antibiotics to restrict their use to prescriptions by certified doctors, and veterinary, and implementation of antibiotic stewardship program [13]. This has created dangerous consequences leading to the “post-antibiotic era” and is challenging with respect to human efforts to overcome it [8].

Zoonosis

Rapid deforestation has led to blurring of boundaries within the ecosystems. The increased contact of humans with animals due to increasing demands for animal products and a decrease in the food biodiversity for animals has led to rise in the incidences of zoonotic infections [14]. Deforestation is indirectly linked to the high incidences of zoonotic diseases in low-middle income countries (LMICs). Various efforts have been advocated to tackle zoonosis. Inter-disciplinary partnership project for Kyasanur Forest Disease (KFD), Western Forest ecosystems in India, Monkey Fever Risk Framing workshop, Bengaluru, India identified key factors likely to play major roles in understanding of emergence and management of KFD [15]. Outreach immunization services in rural settings alongside other public sector field programs were advocated through village and household visits for adequate rabies vaccination coverage. For control of bovine foot‑and‑mouth disease; the Department of Animal Husbandry, Dairying, and Fisheries implemented national vaccination programs [16]. A National Standing Committee on Zoonoses was implemented for the control of zoonotic diseases in India, The Food Safety and Standard Act in India sets limits on toxic materials in natural products and contaminants, levels of residues of antibiotics or pesticides, heavy metals, etc., A manual has also been published by Centre of Zoonosis, National Centre for Disease Control, India [17]. The Sikkim Anti-Rabies and Animal Health (SARAH) program has demonstrated a successful OH model of dog-facilitated rabies elimination [18].

Emerging Re-emerging Diseases

A major public health event occurs every three years, out of which six have already been declared as the Public Health Emergencies of International Concern by the WHO [8]. Emerging diseases pose a grave threat to our community as they increase morbidity and fatality as well as known to have bioterrorism potential [19]. In the past few years, diseases such as SARS, H1N1 influenza, MERS, Nipah virus disease, Ebola hemorrhagic fever and COVID-19 have emerged as massive public health concerns in the form of either outbreaks, epidemics, or pandemics resulting in heavy loss of human life and economic loss [14]. The lack of understanding of the various epidemiological actors due to a weak surveillance system has made the prevention and control of these outbreaks challenging. The Integrated Disease Surveillance Project (IDSP) is committed to working on strengthening of decentralized laboratory-based Information Technology. To prevent and early detecting the emerging diseases causing outbreaks early, the National Institute of Virology (NIV) Pune was reformed [19].

Various efforts are being made to resolve the re-emergence of diseases. To eliminate human anthrax from a tribal district, the OH intervention model of Odisha, India, has been implemented successfully [20]. A trans-sectoral interdependent collaboration between the community, animal sector, private and non-governmental organizations and health sector was implemented as a community engagement activity on dog gestures [21]. A cost-effective data-driven rabies transmission model was developed in Tamil Nadu to address the challenge of rabies [22]. For the control of the Nipah virus outbreak in Kerala a multi-disciplinary Central team from the National Centre for Disease Control, including various cadre of experts in Zoonosis, Epidemiology, Respiratory, and emergency Medicine as well as animal Husbandry, was setup for investigation. Recent deadly outbreaks of Ebola and Zika virus have made one thing clear all epidemics around the world can only be prevented when health systems are well prepared for them [23].

Other Areas

Food safety and water sanitation are yet another important sector of OH approach. Food safety is a shared responsibility and requires intersectoral collaborative initiative through various departments like the food production, the consumers, the food and water quality standard assessors, etc. [24]. A majority of infectious diseases critical to food safety in humans are zoonotic in nature. Foodborne infections like salmonellosis, E. coli, and vibrio species can be transmitted by meat or milk products, as well as fresh vegetables and other environmental sources [6].

Another concern includes an increase in the severity and intensity of droughts and floods are likely to cause alterations in the hydrological cycle. This climate change is likely to result in a shifting of humid and drier forests to different regions. The forests, agriculture, coastal zones as well as natural resources are climate-sensitive sectors that are already facing stress due to socioeconomic pressures. As climate changes, there are increased chances of disease spread due to the expansion of the range of host, agent, and environment hence keeping track of these pathogens is essential [25].

Similarly prioritizing the Wildlife and Veterinary public health (VPH) in India is the need of the hour. The minimizing proximity of humans to wildlife areas to fulfill human needs has made it favorable for diseases to circulate in peri-domestic regions [8]. Wildlife in India has huge public health implications as a large population live close to forest with their livelihoods essentially connected to their ecosystem. In India, animals like cows and monkeys are worshiped and regarded as harmless, but it is also a known fact that primates like monkeys pose risks for humans in the spread of various infections. Migratory birds from the northern Eurasian region have the ability to transmit avian infections like SARS, Psittacosis, borrelia infection, etc. Fruit bats are known reservoirs for the Nipah virus. Rabies is majorly a major disease of mammals like dogs, wolves, foxes, cats, lions, bats, monkeys, etc. Though it is illegal to kill or hunt wildlife animals in India under the Wildlife Protection Act 1972, yet many animals and birds are killed for milk and meat products. In India, VPH assumes a huge significance in developing countries although its implementation has not reached its maturity. Currently, issues in VPH are related to stray animal population, unregulated markets for animal products, no regulations for slaughter in India, animal waste disposal, ethical issues related to animal welfare, zoonotic diseases, need for upgradation of preventive services, quality control in veterinary practice, etc. [26].

A framework for collaboration

Effective implementation of OH needs a framework incorporating policy formation, program implementation, financial support, interdepartmental collaboration, capacity building, and local level participation [2]. Six international organizations: the WHO, FAO, OIE, the United Nations Children’s Fund (UNICEF), the United Nations System Influenza Coordination (UNSIC), and the World Bank joined hands to form a Strategic framework [27].

Implementation and operationalization of OH approach

OH is a complex, sustainable interdisciplinary, practical, and cost-effective approach for developing countries. To support OH collaboration, a regional tripartite coordination was established between the human and animal sectors. This includes workshops for the prevention and control of zoonoses and, the development of tools for the operationalization of OH initiative. At subregional levels, various initiatives for rabies control like the ASEAN rabies meeting, the SAARC rabies meeting, webinars, and workshops on rabies, influenza, and AMR were taken [24]. There is a disparity in shared responsibilities between the central and state government which makes inter-sectoral convergence problematic in nature. However, it is possible to identify and prioritize some important barriers to operationalization and improve the collaboration outcomes of OH. A study based on their findings, suggested the involvement of top-to-down and bottom-to-up governance to overcome the problems and leverage the opportunities for building a functional cross-sectoral OH platform [28]. Various OH/Eco Health Capacity Building (OHEHCB) strategies were implemented in Southeast Asia (SEA) as well as South Asia (SA). A comparison between the two stated that although half of the programs in SEA were short-term, the majority of them were focused on research skills, and targeted to reach a wide range of potential OH personnel like graduates, researchers and programmers, and veterinarians. Overall, there has been a stronger response to OHEHCB in SEA as compared to SA [29]. Some transdisciplinary collaborative initiatives in the various field areas including human and animal health researchers were successfully operationalized (Table 1).

**Table 1:** Examples of some transdisciplinary collaborative initiative in the various fields including human and animal health researchers successfully operationalized

Sr no.	Area of initiative	Collaborations
1.	Research and laboratory collaborations	Tamil Nadu Veterinary and Animal Sciences University (TANUVAS) with the Tamil Nadu Dr MGR Medical University (TNMGRMU) for SARS-CoV2 vaccine
		TANUVAS with Kings Institute of Preventive Medicine and Council of Scientific and Industrial Research Centre for Cellular and Molecular Biology to sequence genomes of SARS-CoV2
		Guru Angad Dev Veterinary and Animal Sciences University (GADVASU) and Dayanand Medical College and Hospital, Ludhiana for testing of Brucella spp.
		Designation of the veterinary labs the National Research Center on Equines, Hissar and Central Military Veterinary Laboratory, Meerut as reference laboratories by MoHFW for the diagnosis of glanders in humans
2.	Disease outbreaks prevention and control	The Indian Council of Medical Research (ICMR)-National Institute of Virology, Pune for KFD and CCHF outbreaks in India
		Directorate of Health Research (MoHFW,), ICAR, State Animal Husbandry, State Health Department and District Administration for the outbreak of Nipah virus
		OH approach being utilized for the control of anthrax in tribal villages of Odisha involving health care sector, animal care sector, NGOs and community social welfare groups
		Veterinarians deployed in isolation wards of hospitals in Haryana state thorugh CO-JEET- victory over COVID-19 extending support to medical personnels
		Cellular and Molecular Biology Center for sequence 21 genomes of SARS-CoV2, TANUVAS partnered with Kings Institute of Preventive Medicine and Council of Scientific and Industrial Research
3.	Community outreach and engagement	GADVASU and Punjab Agricultural University worked with sarpanches of villages for various training and health education programs related to zoonosis,
		Involvement of the village sarpanch, health officers, auxiliary nursing midwives(ANM), and accredited social health activists (ASHA), enabling them to overcome the hesitancy related to the COVID-19 vaccine in Punjab.
		OH centre established by GADVASU engaging communities in training and awareness programs related to zoonossi, AMR, Food and water sanitation and biosecurity measures.
		Farmers FIRST programme, a multidisciplinary team under ICAR and GADVASU and Punjab Agricultural University together with villages for better crop production, pest control and building strong biodiversity.

There is a huge gap regarding awareness of zoonotic disease in the general population. Therefore, there is a need to deliver a zoonotic or OH outreach to the communities, including school children. Engagement of various community health cadres like ASHA workers and Male Multipurpose Health workers from Ahmedabad expressed little interest in working for OH initiatives because of the low financial assistance, recognition, and support [30].

Hence, a need for regional support at the community level was recognized. The Pearl River Declaration, in the SEA-Pacific region, was established to strengthen regional health security and capacity building [31]. A complex system like OH is assigned various actors and their predetermined roles. At the highest level are the administrative actors comprising policy makers, program implementers and planning and management leaders. At the lowest level are community actors consisting of village leaders, health workers, and various community leaders from governmental as well as non-governmental organizations [7]. Robust methodological tools for multicriteria analyses and, a health management system thinking approach have been developed to support the implementation of this approach. A transdisciplinary multicriteria analysis, health management system thinking approach was proposed as one of the robust methodological tools. Similarly, the checklist for reporting of OH Epidemiological Evidence (COHERE) was used to promote the integration of knowledge from the animal, human, and environmental domains [32].

A major part of the control and prevention of various diseases is focused on the early identification of risks followed by early diagnosis and treatment. For this, a "Health system contact" is a better way to provide preventive health care services when a new health need arises. Health System Contact (HSC) can be defined as an individual or person from the health system that can be contacted when a health problem or medical need arises or a person who is providing preventive health care services at the community level. Majorly provided at the community level, male/female health workers are contacted for humans and private veterinarians for animal health [33].

**Figure 2:**
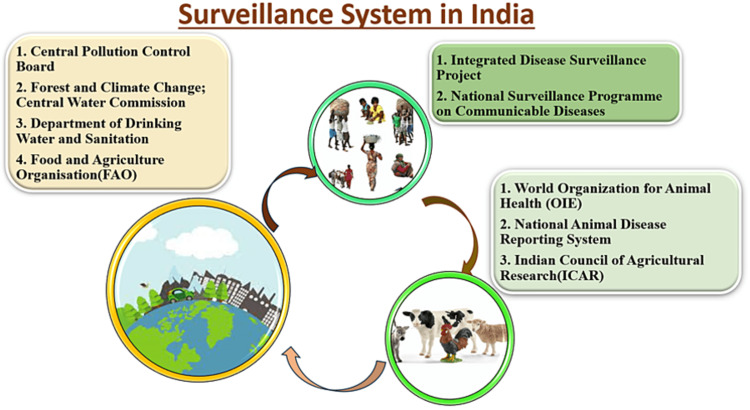
Surveillance system in India (self-made figure)

Surveillance Systems in India consist of three main domains under OH approach as shown in Figure 2. The IDSP for surveillance of humans detects all symptomatic, presumptive, and confirmatory diagnosed cases. The OIE gathers and compiles information on diseases related to livestock and poultry and the Central Pollution Control Board, Forest and Climate Change, Central Water Commission, etc., for monitoring of environmental factors. A recent example of OH surveillance system was boosted during post-COVID-19 times [34].

OH institutional capacity

In India, the disease control system, similar to the healthcare system is Biphasic in nature. The center is accountable for the health regulations and policies related to health and disease, the state is mainly responsible for healthcare and training of health workers at the grassroots level. The infection control strategy in India uses two strategies, first - the vertical programs implemented in programs like RNTCP, NACP, and NVBDCP, etc., and second - the ad-hoc assistance activated on request from the state authority namely the National Center for Disease Control for outbreak investigations and control [35].

At first, the concept of establishing a National Institute for Zoonoses (NIZ) at Nagpur was conceived by the National Association of Welfare of Animals and Research and the Indian Medical Association (IMA). Further the Indian Council of Medical Research (ICMR) jointly with the Indian Council of Agricultural Research (ICAR) Nagpur adopted this idea to create a domineering facility at Maharashtra Animal and Fishery Science University (MAFSU). In 2019, the ICMR declared the establishment of the “Centre for One Health (OH)” in Nagpur under NIV, Pune. Subsequently an independent institute, ‘The National Institute of One Health’ was established under MAFSU in Nagpur by the ICMR and ICAR [36].

OH and India: current challenges

Lack of knowledge - the biggest weakness in developing countries like India is the lack of awareness about zoonotic diseases among animal handlers and the general public. Unavailability of proper records - no prescriptions or hospital records are available with medical professionals and institutions as well. Unpopular veterinarian services among the general public-no practice of obtaining health services for sick animals and no regular checkups and immunizations for animals [4]. Failure of adequate and timely diagnosis and treatment, growing antibiotic resistance among animals, rodents, and insects which persists in the skin and gut, circulating in the ecosystem is yet another challenge. Research gap and evidence gap - very few research have been completely dedicated done on multi-disciplinary OH research. No significant information on zoonotic diseases in humans and animals is available. Lack of training programs - there is a minimal number of capacity building programs, and training for health workers held in the country. Not many long-term education programs for graduates in OH have been implemented in SA. Inadequate government support - few countries have made remarkable progress in OH networking, operationalization of OH, and capacity building while in some countries functionalization of the OH platform has not gained momentum at the regional and national level and is still dependent on donor funding [37].

Key OH strategies in India at different levels and way forward

At the Individual Level

This collaboration takes place between physicians and veterinarians. It helps in assessing risks for individual health and early detection of diseases, e.g., Indian Medical Association, Indian Veterinary Association, OH Clinics, comprehensive primary care through Health and Wellness Centers.

At the Population Level

A collaboration between human and animal healthcare systems during outbreaks e.g. One Health surveillance, integration between two-surveillance systems-human disease, IDSP, and animal disease, NADRS.

At the Research Level

It is between research institutes of humans and animals to form a new scientific insight into disease-causing factors. e.g. ICMR-ICAR/Road map to Combat Zoonosis in India Initiative (RCZI).

A state-specific level-based collaboration strategy for the control of rabies in Tamil Nadu was adopted. The initiatives adopted in our country are either solution-based collaborative or level-based research strategies, e.g., national influenza pandemic committee to control avian influenza and leptospirosis [35]. There are plenty of opportunities for implementing OH in India by increasing the sensitization of the lay public, improving capacity for public health actions, by instituting optimum coordination and collaboration between all stakeholders [4]. The comprehensive table summarizes the important articles' key findings (Table 2).

**Table 2:** Summary of some important articles selected on One Health implementation, operationalization, and challenges in India

Author	Title	Type of Study	Objectives	Key Findings
Dasgupta et al. [1]	Adopting an intersectoral One Health approach in India: Time for One Health Committees	Review Article	Outline the key foundational principles of One Health Committees	Elaborated successful models and domains of OH collaboration and steps to culminate in building OH community networks and promoting the approach.
Sharma [4]	One Health Paradigm: Challenges and Opportunities for Mitigating Vulnerabilities Associated with Health of Living Beings	Editorial	To implement One health systematically through appropriate governance, adequate financing, human resources development, laboratory support and rapid and robust surveillance response systems. Need to create new institutions and centres, effective and fruitful partnerships and networks built and facilitated among the existing institutions for mutual collaboration and joint efforts.	It includes political commitment, policy formulation, sustainable funding, program implementation, institutional collaboration, capacity enhancement, and active participation of the communities forming a part of a systematic approach to the implementation of OH
Kniel et.al [6]	Understanding the Complexities of Food Safety Using a “One Health” Approach	Editorial	To comprehensively address preharvest food safety issues such as AMR and foodborne salmonellosis.	A thorough understanding of the environment and vector reservoirs by all sectors and the need for better One Health research strategies to solve these growing challenges.
Bhatia [8]	Addressing challenges of zoonotic diseases through One Health approach	Perspective	To address the need for urgent, systematic, concrete and multisectoral actions for zoonotic diseases through one health approach.	Need for launching a multi-coordinated global response with active involvement of human, animal and environment health sectors.
Yasobant et al. [9]	Convergence model for effectual prevention and control of zoonotic diseases: a health system study on ‘One Health’ approach in Ahmedabad, India	Study Protocol	To understand the structure and complexity of OH. To provide a system model for describing and enhancing convergence between human and animal health systems.	Expected to develop a system model for enhancing convergence based on the factors that affect the process for effective prevention and control of zoonotic diseases and hence could be a potential source for future One Health policy and planning.
Airikkala-Otter et al. [16]	Sharing the Load by One Health: Integrating Canine Rabies Vaccination With Bovine Foot‑and‑Mouth Vaccination Program and Community Public Health Services in Rural Nilgiris District, Tamil Nadu, India	Short Communication	Evaluate the feasibility of combining canine rabies vaccination with pre-existing animal‑health interventions or public health programs in rural India.	Indicates that vaccination of dogs against rabies can be implemented alongside other public sector field programs, accessing large numbers of dogs in rural settings without specialist dog-catching equipment. Adequate dog rabies vaccination coverage cannot be achieved and maintained in rural India by relying only on fixed-point vaccination centres but requires outreach immunisation services through village and household visits.
Aggarwal et al. [17]	One Health Approach to Address Zoonotic Diseases	Commentary	Addressing the OH approach and several challenges at the level of implementation.	Awareness programs for stakeholders should be increased, a national disease registry of zoonotic diseases needs to be developed, and increased vaccination coverage to combat zoonosis.
Bhattacharya et al. [20]	One Health approach for elimination of human anthrax in a tribal district of Odisha: Study protocol	Study protocol	To describe the implementation and evaluation of the ‘One Health’ intervention model based on the principles of the Theory of Change (toc) to eliminate human anthrax from a tribal district in Odisha, India.	To build a surveillance network to be strengthened to track the cases in early stage and risk zoning will be done for focused surveillance in endemic areas. To provide insights for policy-making their replication in other endemic regions.
Gautam et al. [21]	Multisectoral approach to achieve canine rabies-controlled zone using Intervention Mapping: Preliminary results	Original Article	To develop an intervention strategy, using an Intervention Mapping framework tailored for the target community to achieve a canine rabies-controlled zone.	Lack of participation by the study population for canine vaccination, incomplete knowledge about the annual canine vaccination schedule, lack of understanding of dog gestures, lack of infrastructure and resources at veterinary hospitals
Fitzpatrick et al. [22]	One Health approach to cost-effective rabies control in India	Original article	To implement a data-driven rabies transmission model fit human rabies autopsy data and human rabies surveillance data from Tamil Nadu.	Highly feasible strategies focused on stray dogs, vaccinating 13% of stray dogs could cost-effectively reduce human rabies by almost 90% within 5 years.
Singh et al. [25]	Role of India’s wildlife in the emergence and re-emergence of zoonotic pathogens, risk factors and public health implications	Review article	Indicating the essential role of wild animals in the emergence and re-emergence of zoonotic pathogens and providing brief summaries of the zoonotic diseases that have occurred in wild animals in India.	The dependence of large human populations on forests, climate change, deforestation, expanding human population, and agricultural activities resulted in an increased risk of the emergence and re-emergence of wildlife-related zoonotic pathogens in India.
Gongal [27]	One Health Approach in the South East Asia Region: Opportunities and Challenges	Article	To promote a culture of working together sustainably, particularly in resource-constrained countries, to address health risks at the human–animal interface on an OH platform. Recognise a need for institutional development to operationalise and sustain practical applications of One Health at the ground level with the support of local champions who may be working with government, non-government organisations, and academic institutions.	Political assurance by national governments is fundamental in promoting an OH approach to respond to and manage zoonotic diseases supported through policy decisions.
Asaaga et al. [28]	Operationalising the “One Health” approach in India: facilitators of and barriers to effective cross-sector convergence for zoonoses prevention and control	Systematic review	To address the gap by exploring the facilitators of and barriers to successful convergence between the human, animal and environmental health sectors in India	Limited policy visibility of zoonotic diseases has high recognition in the existing policy agenda setting. Participants identified the need for more supportive policies, conflicting departmental priorities and limited institutional capacities as major barriers that hamper effective cross-sectoral collaboration on zoonotic disease control.
Chatterjee et al. [29]	One Health/eco-health capacity building programs in South and South East Asia: a mixed method rapid systematic review	Systematic review	To conduct a situation analysis of the existing OH/EH capacity-building programs, with a focused analysis of those programs with extensive OH engagement, to help map the current efforts in this area.	SEA has used systematic investment and support to develop the OH/ EH agenda and capacity-building strategies in the core competencies. Strategic funding decisions need to target capacity building in the core OH/EH competencies, especially related to trans-disciplinarity, systems thinking, and adaptive management, to effectively address the disease emergence hotspots in these regions.
Yasobant et al. [33]	Health System Contact and Awareness of Zoonotic Diseases: Can it Serve as One Health Entry Point in the Urban Community of Ahmedabad, India?	Original Article	The comprehensiveness of service delivery, first contact, community orientation, coordination, family-centeredness, cultural competence and awareness of selected zoonotic diseases	Significant differences in the dimensions first contact, coordination, and the family centeredness in the HSC score of both systems are evident. The coordination score could be higher in the animal sector, indicating the scattered service (done by different private actors) rather than a coordinated government-led service for the human health system.
Yasobant et al. [34]	COVID-19 in India: Making a Case for the One Health Surveillance System	Review Article	To boost the existing surveillance activities for early detection and ways to develop an integrated OHS to prevent future pandemics in India. To provide possible solutions at the interface of human–animal–environment, from the simpler to the complex system integration with the principles of one health.	The multidisciplinary approach to risk identification is essential for early detection and containment of future (re) emerging diseases.
Mckenzie et al. [37]	One Health research and training and government support for One Health in South Asia	Review article	To describe OH research and training and capacity building activities and the critical developments in government support for OH in these countries to identify current achievements and gaps.	A small number of multisectoral OH research and OH capacity-building programmes were conducted in the region. Identified gaps were a need for more useful scientific information and a collaborative culture for formulating and implementing integrated zoonotic disease control policies and the need for ongoing support for transdisciplinary OH research and policy-relevant capacity-building programmes.

## Conclusions

The recent dreadful outbreaks have made urgent actions necessary, and a functional multi-collaborating agency is needed to combat situations like the zoonotic pandemic and similar events. In India, where the rearing practices are entangled with cultural beliefs, accepting this approach is provocative. Instead, a step-by-step approach without compromising the human-animal bond should be encouraged by educating the people about the interlinkage between the health of man, animals, plants, and the environment. The focus of the OH activities must be directed towards OH research, OH Training, seeking governmental support for the implementation of various OH health-oriented activities, and institutionalization of OH. It is also necessary to monitor the collaborations through their strength of integration, development as well as the performance of the collaboration. For sustaining collaborations and building resilience, it is important to focus on strengthening the bond between the sectorial collab, which needs time. Expecting immediate outcomes will only diverge the goals to be achieved, while in the long run, we can expect fruitful outcomes leading to a successful collaboration. The main goal of the activities related to OH should be improving early diagnosis and treatment, more focus on prevention and control, and rapid retrieval of the affected species from the disease or outbreak. The recent pandemic of COVID-19 has been a wake-up call for the scientific community & lay people alike. For better preparation for any such Zoonotic pandemic, it is vital that the OH approach, its principles, and determinants are addressed by all the stakeholders responsible for implementing it from the scientific community, medical and veterinary Doctors, Food Industry, Research, and development Units and lay people all work as in sync and as a team approach. Taking cognizance of the OH approach to preventing emerging and reemerging diseases and subsequent catastrophes, interventions at national and international levels have been formulated. Its implementation at the grassroots level addressing all the stakeholders is of paramount importance considering the glocal nature of this approach.
